# Optimization of Chitosan–Alginate Microparticles for Delivery of Mangostins to the Colon Area Using Box–Behnken Experimental Design

**DOI:** 10.3390/ijms21030873

**Published:** 2020-01-29

**Authors:** Kamarza Mulia, Ameninta Cesanina Singarimbun, Elsa Anisa Krisanti

**Affiliations:** Department of Chemical Engineering, Universitas Indonesia, Depok 16424, Indonesia; amecesa@gmail.com (A.C.S.); elsakm@che.ui.ac.id (E.A.K.)

**Keywords:** alginate, Box–Behnken optimization, chitosan, mangostin

## Abstract

Chitosan-alginate microparticles loaded with hydrophobic mangostins present in the mangosteen rind extract have been formulated and optimized for colon-targeted bioactive drug delivery systems. The chitosan–mangostin microparticles were prepared using the ionotropic gelation method with sodium tripolyphosphate as the cross-linking agent of chitosan. The chitosan–mangostin microparticles were then encapsulated in alginate with calcium chloride as the linking agent. The mangostin release profile was optimized using the Box–Behnken design for response surface methodology with three independent variables: (A) chitosan–mangostin microparticle size, (B) alginate:chitosan mass ratio, and (C) concentration of calcium chloride. The following representative equation was obtained: percent cumulative release of mangostins (10 h) = 59.51 − 5.16A + 20.00B − 1.27C − 1.70AB − 5.43AC − 5.04BC + 0.0579A^2^ + 10.25B^2^ + 1.10C^2^. Cumulative release of 97% was obtained under the following optimum condition for microparticle preparation: chitosan–mangosteen particle size < 100 µm, alginate:chitosan mass ratio of 0.5, and calcium chloride concentration of 4% *w*/*v*. The alginate to chitosan mass ratio is the statistically significant variable in the optimization of sequential release profile of mangostins in simulated gastrointestinal fluids. Furthermore, a sufficient amount of alginate is necessary to modify the chitosan microparticles and to achieve a complete release of mangostins. The results of this work indicate that the complete release of mangostins to the colon area can be achieved using the chitosan–alginate microparticles as the bioactive delivery system.

## 1. Introduction

The colon is one of the most important organs in the human digestive tract. Therefore, it is desirable to formulate a colon-targeted drug delivery system to treat various colon diseases such as ulcerative colitis, Crohn’s disease, and colon cancer [1,2]. Patients prefer the oral administration of drugs for the treatment of colonic diseases; however, several factors must be considered first. Colon-targeted orally administered drug delivery systems must be able to protect the drug from the harsh pH conditions in the stomach, and the drug must be released and absorbed in the colon. Inappropriate formulations in combining several polymers as carriers can cause drugs to degrade and fail to cure the disease because they do not reach the location in the colon.

Mangostins from mangosteen peel extract are hydrophobic phenolic compounds that have been identified as strong anti-proliferative agents against human DLD-1 colon cancer cells [3]. Mangostins are reported to show potential as chemopreventive agents for cancer without causing side-effects [4]. Further, mangosteen peel extract was reported to mostly contain α-mangostin and small amounts of β-mangostin and γ-mangostin [5,6,7,8]. Consuming mangostins directly is less effective because these compounds are poorly soluble in aqueous solutions and therefore have low bioavailability. The encapsulation of mangostins in chitosan has been reported to increase their bioavailability and mucoadhesivity [9,10].

A carrier formulated to deliver bioactive compounds to the colon must be resistant to the acidity of gastric acid and to the basicity of the intestine. Therefore, the problem is how to properly select the compounds that is suitable to these conditions. The above mentioned problem can be solved by combining two polymers as a carrier, as the complex formed by the two types of polymers can withstand the acidity of the stomach and the basicity of the intestine and colon [11,12,13]. The carriers (polymers) may affect the release properties and efficacy of the bioactive compounds. The use of polymers that are pH sensitive is preferred because the pH in the digestive system varies from 1.2 in the stomach to 7.4 in the intestine and 6.8 in the colon.

Chitosan, a biopolymer obtained from the shells of crustaceans (i.e., marine animals) [14,15,16], is widely used as a carrier for delivering drugs in the gastrointestinal tract. Chitosan with amino groups is soluble at low pH and insoluble at high pH. It also has good biocompatibility, biodegradability, and nontoxicity, and it can be readily modified for delivering drugs and bioactive compounds [17,18,19,20,21,22]. Owing to these properties, chitosan is widely used in colon-targeted drug delivery formulations. Chitosan can slowly release drugs in the colon [1,23,24].

As the second polymer in the formulation, alginate has been widely chosen as the outermost coating to protect the drugs or bioactive component from the acidity of the stomach. Alginate is a pH-sensitive biopolymer, and it is used widely in drug delivery because of its beneficial properties such as biodegradability, biocompatibility, and nontoxicity [25]. Alginate with carboxyl groups tends to shrink at low pH and dissolve at high pH. This characteristic helps to lower the release of bovine serum albumin [26] from chitosan–alginate microparticles in simulated gastric fluid. In our previous study, the amount of mangostins released from chitosan–alginate microparticles into simulated gastric fluid was found to be lower than that released from chitosan microparticles without alginate. However, in this sequential release experiment, only a small amount of mangostins was released in the simulated colonic fluid over 10–24 h [27]. In another study, alginate microspheres were prepared to reduce the evaporation rate of essential oils via microencapsulation [28].

This study aims to optimize the formulation of chitosan–alginate microparticles as a carrier to deliver mangostin to the colon area such that minimum release is achieved in the stomach but a high and constant release rate is achieved in the colon. First, mangostin-loaded chitosan microparticles were prepared using the ionotropic gelation method with the help of tripolyphosphate as the linking agent. Then, the chitosan–mangostin particles were encapsulated in alginate with calcium chloride (CaCl_2_) as the linking agent for alginate aggregation [26]. The Box–Behnken experimental design was used for optimizing the microparticle formulation to minimize the number of experiments [29,30,31]. The cumulative release of mangostin was the response variable, and the chitosan–mangostin microparticle size, alginate:chitosan mass ratio, and CaCl_2_ concentration were independent variables.

## 2. Results and Discussion

### 2.1. Mangostin Content in Mangosteen Extract

Previously, the use of UV-vis spectrophotometry to quantify total mangostins in the mangosteen rind extract has been validated against the use of a high-performance liquid chromatography (HPLC) apparatus. The amounts of α-mangostin, β-mangostin, and γ-mangostin contained in the ethyl acetate extract were found to be 42.0%, 1.7%, and 9.3%, respectively [5]. In this study, a calibration curve was constructed using α-mangostin as a standard, and the amount of total mangostins was found to be 57.6 ± 2.4% (*w*/*w*).

### 2.2. Encapsulation Efficiency and Loading Capacity of Mangostin in Chitosan Microparticles

The mangostin encapsulation efficiency and loading capacity of the chitosan microparticles were found to be 95.0 ± 2.1% (*w*/*w*) and 3.8 ± 0.1% (*w*/*w*), respectively. To facilitate a consistent comparison between various chitosan–alginate microparticle formulations, the cumulative release calculation is based on the mangostin loading capacity and the mass of the chitosan microparticles used in the preparation step as both were measured accurately.

### 2.3. Cumulative Release of Mangostins from Chitosan-Alginate Microparticles

**Figure 1:**
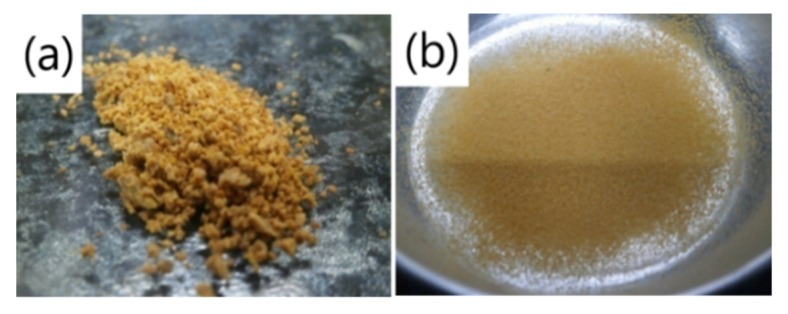
Chitosan–mangostin–alginate dry particles: (**a**) before grinding and (**b**) after grinding with particle size < 100 µm.

**Figure 2:**
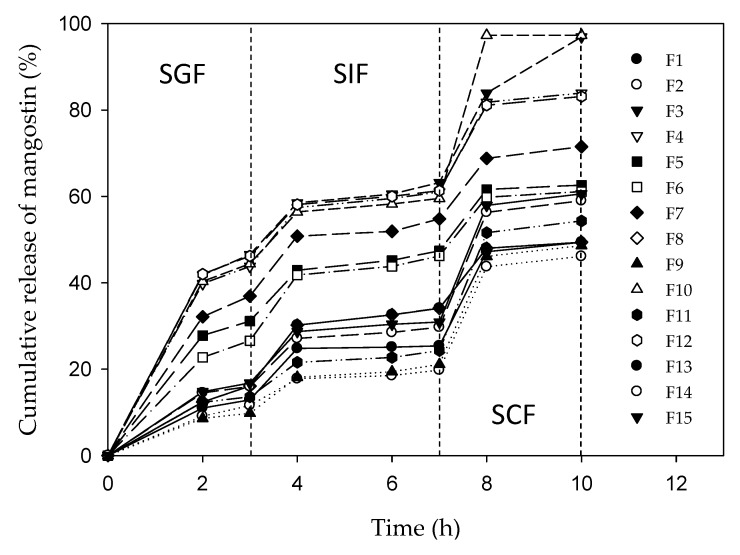
Release profile of alpha mangostin from chitosan–alginate microparticles (0–3 h in simulated gastric fluid (SGF), 3–7 h in simulated intestinal fluid (SIF), and 7–10 h in simulated colonic fluid (SCF)).

Figure 1 shows the chitosan–alginate microparticles optimized for colon-targeted release of mangostins based on the surface response method with Box-Behnken design. Figure 2 shows the experimental cumulative release of mangostins from the chitosan–alginate microparticles immersed sequentially in simulated gastric fluid (SGF) (3 h), simulated intestinal fluid (SIF) (4 h), and simulated colonic fluid (SCF) (3 h). 

Table 1 lists the three independent microparticle preparation variables, each evaluated at three levels. Table 2 shows the experimental and the calculated cumulative release of mangostins (10 h) from 13 microparticle formulas with one triplicate data (F13, F14, F15). A suitable biopolymer formulation could be selected based on different criteria: F10 or F3 for the highest cumulative release at 10 h (97%), F10 or F3 for the most mangostins released in SCF (34–38%), and F2 or F9 for the lowest cumulative release in SGF (9%). Satisfying the first and the third criteria simultaneously is not possible because the cumulative release data at 2 h and 10 h are positively correlated. F2 or F9 might be the desired biopolymer formulation, if, suitable enzymes are present in the colon to catalyze the degradation of chitosan and alginate. Based on the in vitro release data obtained in this work, formulation F10 or F3 is considered optimum for the release of mangostins in the colon because they satisfy the first and the second criteria. These formulations produced microparticles that are prepared with the highest alginate:chitosan ratio of 0.5, in combination with low or medium particle size and calcium chloride concentration. In summary, the chitosan–alginate microparticles could be formulated and optimized for colon-targeted bioactive delivery systems.

### 2.4. Chitosan–Mangostin–Alginate Microparticle Optimization with Box–Behnken Model

Based on the response surface methodology with the Box–Behnken design, the experimental cumulative release data were used to obtain the following cumulative release equation:

%-cumulative release (10 h) = 59.51 − 5.16A + 20.00B − 1.27C − 1.70AB − 5.43AC − 5.04BC + 0.0579A^2^ + 10.25B^2^ + 1.10C^2^ (1)

where A is the size of the chitosan–mangostin microparticles, B is the alginate:chitosan mass ratio, and C is the CaCl_2_ concentration. Table 3 shows the *p*-values of the analysis of variance (ANOVA) that represent the significance of the model and each of the independent variables, as determined using the Design-Expert software. A *p*-value > 0.0001 indicates that the variable has no significant effect on the response.

**Figure 3:**
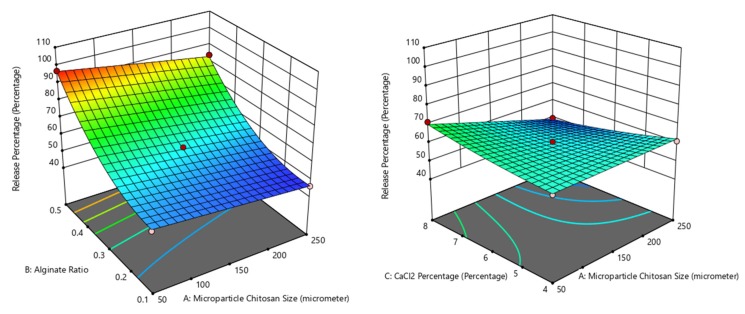
Dependence of mangostin cumulative release on (left) alginate:chitosan ratio and microparticle size and (right) CaCl_2_ concentration and microparticle size.

The Box–Behnken model has a *p*-value < 0.0001; therefore, the interpretation is that the model represents the cumulative release data adequately, consistent with the averaged differences of the calculated and the experimental values of less than 2%. The chitosan:alginate mass ratio is the only statistically significant independent variable with a *p*-value < 0.0001. An examination of the release data, listed from the highest to the lowest value in Table 2, revealed that microparticles with a higher alginate:chitosan mass ratio consistently afford higher cumulative release. Although not statistically significant (*p* = 0.0002), a trend of higher cumulative release afforded by smaller-sized microparticles is observed. The interaction between the alginate:chitosan mass ratio and microparticle size is illustrated by the higher release of F11 (54.3%) compared to that of F8 (49.4%), although the latter formulation has a higher alginate:chitosan mass ratio. Figure 3 shows the effect of the three independent variables on the mangostin release.

### 2.5. Chitosan–Alginate Microparticles as a pH-Sensitive Delivery Formulation

**Figure 4:**
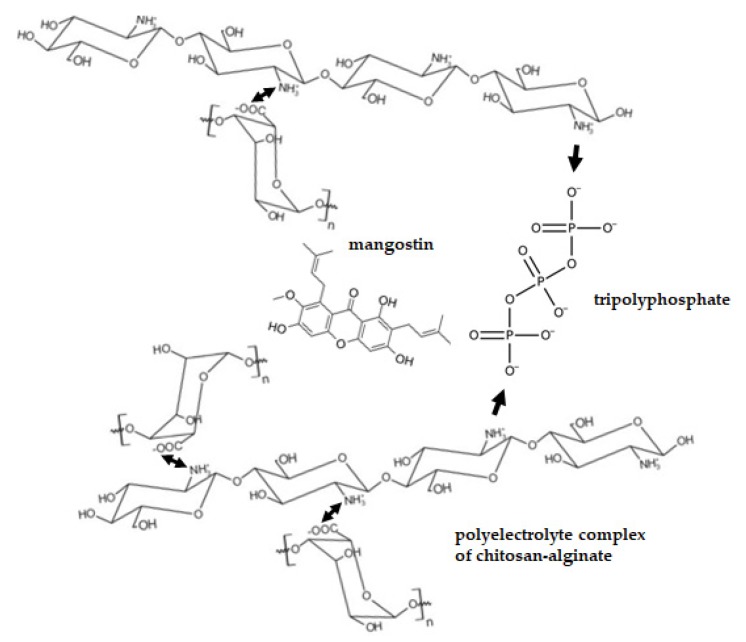
Illustration of the ionically crosslinked chitosan–tripolyphosphate gel (single arrow) and chitosan–alginate polyelectrolyte complex (double arrow) in the chitosan–alginate microparticles.

As alginate droplets are in contact with the CaCl_2_ solution, the calcium alginate complex is concentrated mainly on the outer part of the bead formed. Grinding the beads to less than 100 μm size exposes the chitosan–alginate microparticles to the release media, thereby minimizing the effect of the CaCl_2_ concentration (*p* = 0.0717) and reducing the effect of particle size (*p* = 0.0002) on the amount of mangostins released. Figure 4 shows the conceptualized pH-dependent interactions between components of chitosan–mangostin–alginate microparticles: ionically crosslinked chitosan–tripolyphosphate gel (single arrow) and chitosan–alginate polyelectrolyte complex (double arrow). Mangostins are released mainly in the acidic SGF and in the more neutral SCF, but not to a significant extent in the slightly basic SIF. This pH-sensitive behavior of the chitosan–alginate microparticles is attributed to the pH-dependent nature of chitosan and alginate, in this case, as a polyelectrolyte complex [32].

In acidic SGF, some of the carboxylic groups of the alginate are converted to its acid form and the amino groups of chitosan become protonated. The mangostin release is noted to depend on the characteristics of the chitosan–alginate microparticles or, equivalently, on the amount of alginate that has formed a polyelectrolyte complex with chitosan. The alginate of the polyelectrolyte complex absorbs water and becomes a swollen hydrogel, increases the pore size of the chitosan–alginate microparticles, leading to the release of more mangostins. This explanation is consistent with the fact that the formulations with the highest (F10, F3, F4, and F12) and the lowest (F2, F9, and F1) alginate:chitosan ratio yielded the highest and the lowest cumulative release of mangostins, respectively.

In the slightly basic SIF (pH of 7.4), the release of mangostins is not significant, regardless of the alginate:chitosan ratio. The explanation for this observation is that the amino groups of chitosan are more extensively deprotonated at this pH value, leading to weaker electrostatic repulsion, less swelling, and hindered mangostin release. This observation is in agreement with incomplete release of chlorhexidine acetate in a buffer solution (pH of 7.8) reported previously [33].

In contrast to the flat release profiles obtained in SIF, all formulations released additional mangostins (15–38%) in SCF in a short time interval, between the 7th and 8th hours. As the amount of mangostins released in SIF does not depend on the alginate:chitosan ratio, it seems that the decrease in pH values (7.4 in SIF to 6.8 in the SCF) affects only the amino group of chitosan. There is a limited swelling due to the presence of more deprotonated amino groups, sufficient to induce release of mangostin that maybe reside in the outer layer of the chitosan matrix. After the 8th hour, the cumulative release profiles become flat and mangostins are not released anymore, in agreement with the release profiles of paracetamol in SIF (pH of 7.4), with or without the presence of α-amylase [34]. This finding demonstrates that it is necessary to modify chitosan microparticles with another biopolymer such as alginate, added in a sufficient amount, to achieve complete release of the encapsulated drugs and bioactives.

## 3. Materials and Methods

### 3.1. Chemicals and Materials

Mangostins were extracted from mangosteen rind that was purchased from a local market in East Jakarta, Indonesia. Ethanol 96% (C_2_H_5_OH), ethyl acetate (C_4_H_8_O_2_), acetic acid (CH_3_COOH), sodium tripolyphosphate (STPP), calcium chloride (CaCl_2_), and sodium alginate were purchased from Sigma-Aldrich, Singapore. α-Mangostin 98% as a standard was purchased from Aktin Chemicals, Chengdu, China. Chitosan (medical grade) with a deacetylation degree of 93% and medium molecular weight of 80–120 kDa was obtained from Chemultiguna, Indramayu, Indonesia.

### 3.2. Mangosteen Rind Extraction

Mangosteen rind was cut and washed with Aqua DM. Mangostins were extracted from the mangosteen rind using maceration methods in 96% ethanol with weight:volume ratio of 1:3 (g/mL) and incubated at 50 °C for 2 h [10]. This process was carried out twice. The filtrate from each process was mixed, ethanol was evaporated, and the filtrate obtained was fractionated using an equivolume mixture of ethyl acetate and water. The solvent of the ethyl acetate fraction was evaporated, and the solid extract was stored in a tightly closed bottle.

### 3.3. Quantification of Mangostins

The mangostins in the mangosteen rind extract and in the chitosan–mangostin microparticles were determined quantitatively using a UV-vis spectrophotometer set to measure absorbance at 316 nm [5]. Analytical grade α-mangostin powder (96% purity) was used as a standard for preparing the calibration curve for both analyses.

### 3.4. Preparation of Chitosan–Mangostin–Alginate Microparticles

The chitosan–alginate microparticles loaded with mangostin from mangosteen rind extract were prepared by following previously reported procedures [26,27,28]. A chitosan-extract mixture with mass ratio of 1:0.1 was dissolved in 50 mL of 2.5% (*v*/*v*) acetic acid solution using a mixer set at a speed of 1000 rpm for 15 min. The chitosan-extract solution was dripped slowly using a 2.5 mL syringe into a glass beaker containing 100 mL of 1% (g/mL) tripolyphosphate solution. After waiting for 30 min, the beads formed were collected, washed with aquaDM, and lyophilized using a freeze dryer (EYELA FDV-1200; 47.6 °C; 11.1 Pa) for 24 h. The dried chitosan-mangostin beads were crushed and sieved into several size ranges (< 100 μm, 100–200 μm, and 200–300 μm).

Alginate solution was prepared and mixed with the chitosan–mangostin particles to obtain microparticle suspensions having chitosan:alginate mass ratios of 1:0.1, 1:0.25, and 1:0.5. The suspension was homogenized by mixing at 1000 rpm for 15 min. Then, chitosan–mangostin–alginate beads were formed by dripping the suspension from a 2.5 mL syringe into CaCl_2_ solutions having different concentrations of 4%, 6%, and 8% (*w*/*v*). After waiting for 15 min, the beads formed were collected, washed with AquaDM, and lyophilized using a freeze dryer for 24 h. The dried chitosan–mangostin–alginate beads were then crushed and filtered to produce microparticles with size below 100 μm for use in the release test.

### 3.5. Encapsulation Efficiency and Loading Capacity of Chitosan

The microparticles were characterized in terms of the encapsulation efficiency and the loading capacity, respectively calculated using Equations (2) and (3):

Encapsulation efficiency = mass of mangostin in microparticles/mass of initial mangostin × 100(2)

Loading capacity = mass of mangostin in microparticles/mass of microparticles × 100(3)

The mass of mangostin encapsulated in the chitosan–alginate microparticles can be calculated by subtracting the amount of mangostin found in the filtrate from leaching chitosan–alginate particles from the initial content of mangostin in the extract added to the chitosan–alginate mixture.

### 3.6. In Vitro Drug Release Study

In vitro release was carried out by immersing 20 mg of microparticles in 60 mL of simulated gastrointestinal fluids: simulated gastric fluid (SGF), simulated intestinal fluid (SIF), and simulated colonic fluid (SCF). The simulated gastrointestinal fluids were prepared using 0.2 M KCl and 0.2 M HCl in a volume ratio of 1:1.7 (SGF, pH of 1.2), 0.1 M KH_2_PO_4_ and 0.1 M NaOH in a volume ratio of 1:0.782 (SIF, pH of 7.4), and 0.1 M KH_2_PO_4_ and 0.1 M NaOH in a volume ratio of 1:0.448 (SCF, pH of 6.8). Each batch of microparticles was sequentially immersed in SGF (3 h), SIF (4 h), and SCF (3 h), for a total release time of 10 h. Samples were taken at appropriate times to determine the cumulative release of α-mangostin using a UV spectrophotometric analysis, and the data were plotted as a function of time to obtain the release profiles.

### 3.7. Box–Behnken Experimental Design

The microparticle formulation was optimized using the Box–Behnken design applied to three independent variables, each evaluated at three levels [35]. This experimental design requires evaluation of 15 data points to generate the response surfaces, consisting of a set of points at the midpoint of each edge and the triplicated center point of a three-dimensional cube. A second-order polynomial equation (Equation (4)) was used to fit the experimental data:

(4) $$Y=\beta_{0}+\overset{k}{\underset{j=1}{\sum }}\beta_{j}X_{j}+\overset{k}{\underset{j=1}{\sum }}\beta_{jj}X_{j}^{2}+\underset{i}{\sum }\overset{k}{\underset{j>i}{\sum }}\beta_{ij}X_{i}X_{j}+e_{i}$$

where *Y* is the response variable; *X_i_* and *X_j_* are independent variables; *β*_0_ is the model intercept coefficient; *β_j_*, *β_jj_*, and *β_ij_* are the interaction coefficients of the linear, quadratic, and second-order terms, respectively; *k* is the number of independent parameters; and *e_i_* is the random error.

The effect of each variable on the cumulative release of mangostin was obtained using the Design-Expert software. Statistical comparison was performed using one-way analysis of variance (ANOVA). The response variable in this study is the cumulative release of mangostin (*Y*), and it depends on the following independent variables (*X*): (A) chitosan–mangostin microparticle size, (B) alginate:chitosan mass ratio, and (C) CaCl_2_ concentration. Each variable was divided into three levels: low (−1), medium (0), and high (+1) as listed in Table 1. The levels of microparticle size and CaCl_2_ concentration were estimated to provide reasonable release of mangostins, based on the release of bovine serum albumin from chitosan–alginate–pectin microparticles [26]. The highest alginate:chitosan mass ratio was set to 0.5 to maintain the mangostins loading capacity of the microparticles and to maintain the desirable characteristics of chitosan in terms of bioavailability and mucoadhesivity [9,10].

## 4. Conclusions

A chitosan–alginate microparticle formulation was prepared as a mangostin carrier, and its release in simulated gastrointestinal fluid was studied. Optimization using the surface response methodology and Box–Behnken Design with Expert-Design Software resulted in formulations with cumulative release of 97%. These microparticles were prepared using the highest alginate:chitosan ratio of 0.5 in combination with low or medium particle size and calcium chloride concentration. The *p*-value indicates that the alginate:chitosan mass ratio in the microparticle formulation is the statistically significant variable affecting mangostin release from the microparticles in the simulated gastrointestinal fluids. A sufficient amount of alginate is necessary to modify the chitosan microparticles and to achieve the complete release of mangostins.

## Figures and Tables

**Table 1:** Levels of the independent variables.

Level	Microparticle Size (µm) (A)	Alginate: Chitosan Mass Ratio (B)	CaCl_2_ Concentration (% *w*/*v*) (C)
Low (−1)	<100	0.10	4
Medium (0)	100–199	0.25	6
High (1)	200–299	0.50	8

**Table 2:** Microparticle formula, coded level, and mangostin cumulative release at 10 h.

Formula	Microparticle Size in µm (A)	Alginate: Chitosan Mass Ratio (B)	[CaCl_2_] in %-*w*/*v* (C)	Cumulative Release (%)	
				expt.	calc.
F10	0	1	−1	97.5	97.2
F3	−1	1	0	96.9	96.7
F4	1	1	0	83.9	83.0
F12	0	1	1	83.0	84.5
F7	−1	0	1	71.3	70.0
F5	−1	0	−1	61.1	61.7
F6	1	0	−1	60.9	62.2
F15	0	0	0	59.0	59.5
F13	0	0	0	60.6	59.5
F14	0	0	0	58.9	59.5
F11	0	−1	1	54.3	54.6
F8	1	0	1	49.4	48.8
F1	−1	−1	0	52.3	53.3
F9	0	−1	−1	48.6	47.1
F2	1	−1	0	46.1	46.4

**Table 3:** *p*-values of ANOVA for Box–Behnken model.

Source	*p*-Value
Model	<0.0001
Microparticle size	0.0002
Alginate:chitosan mass ratio	<0.0001
CaCl_2_ concentration	0.0717
